# Nicorandil, Novel Potassium Adenosine Triphosphate Channel (K+ ATP) Opener in Ischemia With Non-Obstructive Coronary Arteries (INOCA) and Atherosclerotic Cardiovascular Disease (ASCVD): Delphi Consensus Statement

**DOI:** 10.7759/cureus.33624

**Published:** 2023-01-10

**Authors:** Rabin Chakraborty

**Affiliations:** 1 Cardiology, Medica Superspecialty Hospital, Kolkata, IND

**Keywords:** type 2 diabetes, nicorandil, microvascular angina, angina non-obstructive coronary artery disease, ischemic non-obstructive coronary artery disease, coronary artery disease

## Abstract

Background: Coronary artery disease (CAD) is a significant health concern today that has assumed epidemic proportions worldwide and in India.

Objective: The pathobiology of CAD or ischemia with non-obstructive coronary arteries disease (INOCA) in most cases involves coronary microvascular dysfunction (CMD) that often requires personalized pheno-approach based on the risk stratification and diagnostic yield, especially in patients with recurrent angina episodes. Chronic therapy with anti-anginal is often fraught with challenges of unipolar action, tolerance, and oxidative stress that precludes consistent benefits in clinical cases of CMD represented by microvascular angina (MVA). To further elucidate the clinical role and relevance of long-term vasodilator therapy, including nicorandil, a Knowledge, Attitude, and Perception (KAP) survey was planned to be conducted.

Methods: Based on responses to the KAP survey questionnaire, Delphi-mediated graded recommendations were developed by a specialist panel as per Agency of Health Care and Quality Systems (AHRQ) criteria.

Results: Management of INOCA with MVA requires a multidisciplinary approach involving non- or invasive procedures that may be relevant for persistent or refractory angina before instituting symptomatic and long-term use of nicorandil for beta-blocker intolerant and associated clinical phenotypes.

Conclusion: The analyses provide a real-life approach to the management of INOCA and angina non-obstructive coronary artery disease (ANOCA) with underlying CMD that may be relevant in outpatient settings in India.

## Introduction

Cardiovascular disease (CVD) related to coronary arteries is a leading cause of morbidity and mortality in India and worldwide.

The prevalence of CVD, including coronary artery disease (CAD), has significantly increased in the last three decades in India. The majority is higher in urban areas at 2.5-12.6% compared to 1.4-4.6% in rural areas. The systemic review of Indians with CAD further highlighted the prevalence of risk factors such as smoking (8.9-40.5%), hypertension (13.1-36.9%), and diabetes mellitus (0.2-24.0%). In most cases, the door-to-needle time after myocardial infarction was >300 mins, while the discharge medications were skewed towards anti-platelets, beta-blockers, and renin-angiotensin-aldosterone system (RAAS) inhibitors [1] with little credence to anti-anginal for consistent therapy in such cases of chronic coronary syndromes (CCS) or clinical phenotypes including ischemia with non-obstructive coronary arteries (INOCA).

The ignominy of CAD is further highlighted by the early induction almost a decade earlier than the developed countries with rapid progression and high mortality rates. The projected data show that from 1990 to 2020, there will be a 117% and 105% rise in mortality from CAD in men and women, respectively, in India, thanks to the growing population and aging, a risk determinant.

Indians are liable to get hospitalized two to four times more frequently for complications of CAD compared with other ethnic groups, and admission rates are five to ten times higher for populations younger than 40 years which seems to have little correlation with the concomitant prevailing or associated risk factors. The prevalence of CAD in Indians, however, is around 21.4% for diabetics and 11% for non-diabetics [2] with onward endothelial distress-related complications or diastolic dysfunction.

Ischemia with non-obstructive coronary arteries (INOCA)

The diagnosis and management of CAD remain a challenge with recent awareness of several phenotypes, including INOCA. Nearly 30% to 70% of patients with angina and signs of ischemia referred to coronary angiography have non-obstructive coronary artery disease [3]. INOCA has been diagnosed in the absence of coronary artery stenosis >50% degree of stenosis (DS) or with a fractional flow reserve (FFR) <0.80 [4], which may require consistent therapy beyond the conventional standard of care for the risk factors since they are pretty characterized by coronary microvascular dysfunction (CMD) that is often associated with worse clinical outcome and course with recurrent angina episodes.

In most of these cases, there is an underlying CMD associated with subclinical stenosis and spasm that is heralded by the following phenotypes: Chronic stable angina (CSA) with persistent angina despite revascularization; Rest angina with persistence due to associated vasospasm with microvascular angina (MVA); Atypical angina in smokers, diabetics, women, and elderly; idiopathic dilated cardiomyopathy with chronic diastolic or systolic failure.

INOCA or MVA: Diagnosis

Definite clinical diagnosis of MVA is challenging, with invasive or noninvasive methods for investigation often warranted in most cases. However, the electrocardiogram may be unremarkable, with Transthoracic Doppler echocardiography able to evaluate the left anterior descending coronary artery only in some patients. Radio-isotope imaging can detect only severe localized disease. Noninvasive diagnosis needs a high index of suspicion. Definite diagnosis is usually based on documentation of near-normal epicardial coronaries with coronary flow reserve < 2.5. Intravascular ultrasound (IVUS) and optical coherence tomography (OCT) can delineate coronary narrowing that is mild yet significant to cause secondary ischemia due to arrhythmia or anemia; however, cardiac positron emission tomography (PET) is increasingly advocated and utilized for the diagnosis as a noninvasive strategy.

INOCA and guideline-directed medical therapy (GDMT)

In the *EAPCI Expert Consensus Document on Ischaemia with No Obstructive Coronary Arteries in Collaboration with the European Society of Cardiology Working Group on Coronary Pathophysiology & Microcirculation Endorsed by Coronary Vasomotor Disorders International Study Group*, the role of CCs, ARBs, beta-blockers, anti-ischemic drugs, and long-acting vasodilators including nicorandil was well highlighted for its impact on coronary microvasculature [4].

The clinical endotypes of CAD (i.e., INOCA) and heart failure as MVA with or without vascular spasm have evolved to suggest the clinical role of nicorandil as appropriate with a statin, trimetazidine, and beta-blockers.

The European Society of Cardiology (ESC) 2019 Chronic Coronary Syndromes (CCS) guidelines recommend an algorithmic approach to anti-anginal drug selection and choice, taking into consideration the updated scientific evidence with complementary mechanisms that may hold relevance especially risk-stratified cases of CAD with polyvascular disease or type 2 diabetes (T2D). Patients with T2D have diffuse and extensive CAD with CMD that may warrant a consistent use of anti-anginal [5]. 

In this line, to further assess the scope, potential, and current contemporary practices towards the long-term management of INOCA in real-world settings of India with long-acting vasodilators and nicorandil, a Knowledge, Attitude, and Perception (KAP) survey was planned to be conducted.

## Materials and methods

A KAP survey questionnaire was developed to discuss some pertinent issues on the management of INOCA with particular emphasis on patients with T2D, poly-vascular disease, residual ischemic risk, and persistent angina in the Indian context. This questionnaire was validated by the three-membered multidisciplinary panel of eminent interventional and practicing cardiologists and endocrinologists with national academic and clinical standing and relevant experience developing consensus statements. The KAP survey was shared electronically with a 13 membered distinguished panel of interventional cardiologists from different centers of excellence in India before its analyses and consolidation during the physical meet conducted in July 2022. During the meeting, recommendations were evolved based on the Quality of Evidence using Agency of Health Care and Quality Systems (AHRQ) criteria with general expert opinion statements based on cumulative experience shared by the panel.

Ethics approval

This study was conducted as a national meeting involving healthcare professionals (HCPs) across the country to reflect on the current insights, practices, and attitudes toward patient demographic or characteristics that hold relevance for intervention with the current standard of care or evolving therapies according to the principles of Helsinki Declaration.

A systemic review of the literature was conducted to collate recent information or articles using PubMed, Google Scholar, and Cochrane Database of Systematic Reviews to search for the highest level of evidence (LoE) relating to each recommendation from their date of initiation to July 2022. The relevant citations and their abstracts were reviewed by the panel who used the information before assigning graded recommendations based on the level, strength, and consistency of evidence while developing opinions on the appropriate use of vasodilators and nicorandil for various clinical conditions and phenotypes in case they were not available.

As applicable in appropriate cases, these graded recommendations were developed as per the methodological stratification as meta-analyses, randomized, and observational studies as type and LoE: (I) Meta-analysis of multiple well-designed randomized controlled studies; (II) At least one well-designed experimental or randomized controlled study; (III) Well-designed, quasi-experimental, longitudinal case-cohort studies with observational design; (IV) Well-designed, non-experimental, cross-sectional, case-control studies; (V) Case series and reports; Strength and consistency of evidence: (A) There is evidence of type I or consistent findings from multiple studies of type II, III, or IV. (B) Evidence of type II, III, or IV, with consistent results. (C) There is evidence of type II, III, or IV, with inconsistent results on safety or efficacy. (D) There is only type V evidence as expert opinion or case report.

The expert opinion was accepted as a recommendation in case of acceptance by 80% of the panelist based on a 3-point LIKERT scale score (1: Disagree; 2: Neutral; 3: Agree) conducted during the meeting.

Descriptive statistics for mean, median, and proportion analyses were carried out for each response generated for the 9-point questionnaire.

## Results

Thirteen HCPs as Interventional Cardiologists provided clinical insights on the structured questionnaire related to the contemporary positioning of long-acting vasodilators, including nicorandil as parenteral/oral formulations in the management of INOCA, unstable angina, and revascularization procedures including PCI using structured KAP based questionnaire. Responses were collated and analyzed by HCPs from different geographic locations across all India.

The structured questionnaire with the responses is highlighted in Table 1.

**Table 1:** Knowledge, Attitudes, and Perception survey and responses on the clinical use of vasodilators including nicorandil in angina and CCS LAN: long-acting nitrates; INOCA: ischemia with non-obstructive coronary arteries; MVA: microvascular angina; CCS: chronic coronary syndrome; PCI: percutaneous coronary intervention; PTCA: percutaneous transluminal coronary angioplasty

No.	Structured questionnaire	Response	%
1	In your opinion are Long-acting vasodilators (LAN or Nicorandil) underutilized in Chronic Stable Angina?	A. Disagree	18%
		B. Neutral	9%
		C. Agree	73%
2	Long-acting vasodilators (LAN or Nicorandil) can be recommended for INOCA. Do you agree?	A. Disagree	0%
		B. Neutral	9%
		C. Agree	91%
3	In patients of INOCA with MVA and tachycardia, what is the ideal therapy?	A. BB+ Nicorandil+ Ivabradine	8%
		B. BB + Nicorandil	54%
		C. BB + Nicorandil + Trimetazidine	38%
		D. None	0%
4	Nicorandil can be recommended in CCS cases for associated left ventricular dysfunction and coronary artery diffuse disease. What is your opinion?	A. Disagree	0%
		B. Neutral	0%
		C. Agree	100%
		D. Not Applicable	0%
5	The suggested use of Vasodilators including Nicorandil for CCS or INOCA with _________	A. Microvascular dysfunction	26%
		B. Vasospastic angina (VSA)	32%
		C. MVD + VSA	42%
		D. Not applicable	0%
6	Peri-PCI intravenous Nicorandil preserves cardiac function due to _________	A. Afterload reduction	20%
		B. Preload reduction	0%
		C. Preload and Afterload reduction	60%
		D. None of the above	20%
7	What is the place in therapy for Nicorandil Injection?	A. No Reflow phenomenon	17%
		B. Coronary Slow Flow	22%
		C. Polyvascular coronary artery disease with High ischemic risk	22%
		D. Management of Unstable Angina	39%
		E. None of the above	0%
8	As per your clinical experience, in Post-PCI settings with any of the risk typifying characteristics, how long do you recommend oral Nicorandil use following IV formulation?	A. 3 months	56%
		B. 6 months	0%
		C. 12 months	22%
		D. Do not use oral Nicorandil after IV	22%
9	How many of your PTCA cases are likely to be INOCA?	A. <10%	27%
		B. 10-20%	55%
		C. 21-40%	18%
		D. 41-60%	0%

## Discussion

INOCA or MVA remains a clinical enigma from diagnosis to therapy that is well-highlighted by persistent or recurrent symptoms in most cases. These symptoms may be explained by ongoing ischemia secondary to CMD that may warrant the consistent use of vasodilators or nicorandil.

Nicorandil: Coronary microvessel dilator

Mitochondrial dysfunction plays a central role in arterio-venular vasodilation and myocardial reperfusion injury. Preclinical studies have illustrated that nicorandil modulates the mitochondrial KATP (mito-[K+]ATP) channels to alleviate endothelial dysfunction while alleviating further reduction in coronary (macro) and capillary (micro) vascular bed resistance leading that avoiding no-reflow or slow flow phenomenon in ischemia-reperfusion injury models [6].

**Figure 1:**
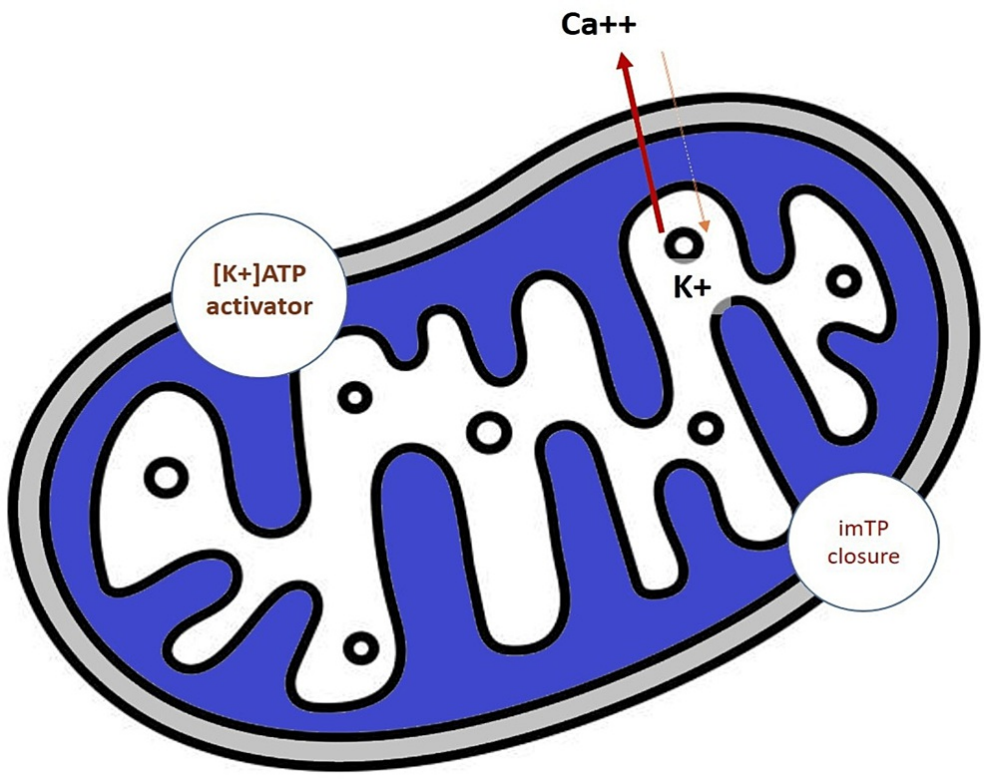
Coronary microcirculatory defect and vasodilation with nicorandil [K+]ATP: Potassium Adenosine Tri-phosphate channel; imTP: Inner mitochondrial membrane transition pore

Anti-anginal drugs, including Nicorandil, might contribute to more favorable acute clinical outcomes due to the inhibition of platelet aggregation and other antithrombotic and anti-inflammatory effects. In addition, nicorandil closes the inner mitochondrial membrane pore to maintain an energy gradient in myocardial ischemic tissues to limit the infarct size or reperfusion injury (Figure 1).

The multimodal action of nicorandil as a K+ ATP channel activator offers arterio-venular dilation at therapeutic dosages while correcting microvascular dysfunction, unlike other long-acting nitrates (Table 2).

**Table 2:** Vasodilators as long-acting nitrates and nicorandil in the management of ASCVD RCT: Randomized controlled trial; ASCVD: Atherosclerotic cardiovascular disease; [K+]ATP: Potassium Adenosine Tri-phosphate channel

Sr	Parameter	Long-acting nitrates	Nicorandil
1	Mechanism of Action	Nitric Oxide	Multimodal as Nitric Oxide donor and [K+]ATP channel agonist
2	Mechanistic for Microvessel dilation	No	Microvessel dilator that avoids the 'STEAL' phenomenon
3	Clinical evidence (i.e. RCT) in ASCVD cases	Yes	Yes
4	Clinical benefit as an Add-on strategy	Yes	Yes
5	Safety issues on Tolerance and Endothelial dysfunction	Yes	No

Nicorandil: Clinical evidence

Nicorandil as oral or IV/IC in offers differential and unique action as a K+ ATP opener for a potential clinical role in CSA and acute coronary syndrome (ACS) requiring percutaneous coronary intervention (PCI), unlike long-acting nitrates [7], including for cases of refractory angina [8].

Nicorandil is a clinically effective recommended option for managing vasospastic angina [9,10].

Nicorandil, in addition, may be beneficial in patients with primary CMD, as highlighted by clinical studies in cases of cardiac syndrome X and MVA [11,12].

Nicorandil, as an intravenous or intracoronary injection followed by an oral strategy, has been shown to improve flow-related CMD as chronic slow flow or no-reflow in CSA or ST-elevation myocardial infarction (STEMI) cases undergoing PCI [13-17]. A meta-analysis involving 24 trials involving 2965 patients with acute myocardial infarction (AMI) showed that nicorandil treatment significantly suppressed the incidence of the no-reflow phenomenon and reperfusion arrhythmia after reperfusion, improved the left ventricular ejection fraction and left ventricular end-systolic volume index, and reduced major adverse cardiovascular events and cardiovascular death when administered for 29 days [18] when inducted as initial-line therapy.

In the Osaka Acute Coronary Insufficiency Study (OACIS) registry, 1846 consecutive patients with multivessel disease, in most cases with recent AMI undergoing emergency PCI, were evaluated for the clinical impact of nicorandil. Of these patients, 535 were treated with oral nicorandil at discharge, while the remaining 1311 patients did not receive nicorandil at discharge. Nicorandil treatment was associated with a nearly 50% reduction in all-cause mortality following release for AMI (HR 0.495, 95% CI: 0.254-0.966, p = 0.0393) [19].

A nationally representative panel of experts responded and reviewed the literature involving randomized controlled trials for vasodilators and nicorandil before developing consensus on their clinical role and positioning in the management of ASCVD (including INOCA) and ACS requiring PCI. The LoE and LoA were developed after a review of literature and publications featuring in indexed databases of Medline, PubMed, SCOPUS, INDEX MEDICUS, and COCHRANE Systemic reviews using the keywords as Ischemic non-obstructive coronary artery disease, angina with non-obstructive coronary arteries, Microvascular angina, Nicorandil, Chronic coronary syndrome, Type 2 diabetes.

The panel evolved a set of clinical recommendations to evaluate the role and relevance of vasodilators and nicorandil in ASCVD, including INOCA that was validated with the strength of evidence with the level of agreement (LoA) reached with each of the members (Table 3).

**Table 3:** Clinical recommendations on the use of vasodilators and nicorandil in angina and CCS CAD: coronary artery disease; FFR: fractional flow reserve; CFR: coronary flow reserve; CMR: cardiac magnetic resonance; NRP: no reflow phenomenon; STEMI: ST-elevation myocardial infarction; CSF: chronic slow flow; ANOCA: angina non-obstructive coronary artery disease; MVD: microvascular dysfunction; CCS: chronic coronary syndrome; T2D: type 2 diabetes; AMI: acute myocardial infarction; EKG: electrocardiogram; LoE: level of evidence; LoA: level of agreement

Sr.	Recommendations	Level & Strength of Evidence or Expert opinion
1	CAD cases with Refractory angina with likely Microvascular angina may be investigated with Biomarkers (Troponin T) and EKG before undergoing elective PCI	Expert Opinion; LoA: 90.9%
2	The use of FFR/CFR and CMR is recommended for CAD or INOCA with recurrent angina episodes.	Expert Opinion, LoA: 90.9%
3	The clinical suspicion of MVD with microthrombi or embolization should be high in T2D or cases with polyvascular disease despite central artery stenosis as <50%	Expert opinion; LoA: 100%
4	In Post-PCI settings for cases with STEMI, parenteral nicorandil can be switched to oral strategy for clinical patients with documented CMD, ie. NRP or CSF phenomenon	LoE: IIA
5	In Post-PCI settings for cases with AMI, parenteral nicorandil can be switched to oral strategy as long-term therapy to improve coronary flow/function while avoiding NRP or myocardial reperfusion injury in the first four weeks of treatment.	LoE: IIA
6	Oral nicorandil can be suggested in cases with refractory angina or persistent symptoms despite the use of nitrates.	LoE: IIIA
7	In INOCA cases undergoing PCI, long-term therapy with oral nicorandil can be suggested to avoid complications, especially in recurrent cases with risk traits of polyvascular disease, hypertension, and age <75y	Expert opinion, LoA: 100%
8	The addition of Nicorandil to Nitrates for complementary actions in CCS cases remains debatable, with low or insufficient evidence for any long-term outcomes or benefits.	LoE: IIC

Limitations

This analysis is limited by the interpretation of the evidence categorized as experimental and real-life observational studies as registries conducted with a heterogenous patient population of CAD and ACS cases requiring PCI with a coronary stent.

## Conclusions

The analyses provide a real-world approach to the management of INOCA with nicorandil as initial-line therapy. GDMT involving nicorandil in post-PCI settings may be relevant in cases of ASCVD with persistent angina or with demonstrable or suspected instances of chronic slow flow (CSF) during cardiac catheterization. The risk characterization for CSF requiring consistent therapy with nicorandil includes local ethnicity traits of small coronary architecture, T2D, polyvascular disease, and high coronary artery calcium (CAC) scores in most cases of ASCVD.
